# Ion Doping Effects on the Lattice Distortion and Interlayer Mismatch of Aurivillius-Type Bismuth Titanate Compounds

**DOI:** 10.3390/ma11050821

**Published:** 2018-05-17

**Authors:** Yu Chen, Jiageng Xu, Shaoxiong Xie, Zhi Tan, Rui Nie, Zhongwei Guan, Qingyuan Wang, Jianguo Zhu

**Affiliations:** 1 School of Mechanical Engineering, Chengdu University, Chengdu 610106, China; chenyuer20023@163.com; 2 College of Materials Science and Engineering, Sichuan University, Chengdu 610065, China; zhi.tan@uta.edu (Z.T.); nierui129@163.com (R.N.); 3 School of Architecture and Civil Engineering, Chengdu University, Chengdu 610106, China; xxujiageng@163.com; 4 College of Architecture and Environment, Sichuan University, Chengdu 610065, China; xsxdyx@126.com; 5 School of Engineering, University of Liverpool, Liverpool L69 3GQ, UK; zguan@liv.edu.uk

**Keywords:** Bi_4_Ti_3_O_12_, lattice distortion, interlayer mismatch, oxygen-octahedron, elastic model

## Abstract

Taking Bismuth Titanate (Bi_4_Ti_3_O_12_) as a Aurivillius-type compound with *m* = 3 for example, the ion (W^6+^/Cr^3+^) doping effect on the lattice distortion and interlayer mismatch of Bi_4_Ti_3_O_12_ structure were investigated by stress analysis, based on an elastic model. Since oxygen-octahedron rotates in the ab-plane, and inclines away from the c-axis, a lattice model for describing the status change of oxygen-octahedron was built according to the substituting mechanism of W^6+^/Cr^3+^ for Ti^4+^, which was used to investigate the variation of orthorhombic distortion degree (*a/b*) of Bi_4_Ti_3_O_12_ with the doping content. The analysis shows that the incorporation of W^6+^/Cr^3+^ into Bi_4_Ti_3_O_12_ tends to relieve the distortion of pseudo-perovskite layer, which also helps it to become more stiff. Since the bismuth-oxide layer expands while the pseudo-perovskite layer tightens, an analytic model for the plane stress distribution in the crystal lattice of Bi_4_Ti_3_O_12_ was developed from the constitutive relationship of alternating layer structure. The calculations reveal that the structural mismatch of Bi_4_Ti_3_O_12_ is constrained in the ab-plane of a unit cell, since both the interlayer mismatch degree and the total strain energy vary with the doping content in a similar trend to the lattice parameters of ab-plane.

## 1. Introduction

Fifty years ago, Aurivillius [1,2,3] discovered a family of bismuth compounds with a layer structure, and to date, more than 50 compounds are known to belong to this group. As most of them were found to be ferroelectric, this group was therefore called Bismuth Layer-Structured Ferroelectrics (BLSF), or Aurivillius compounds. In recent years, BLSFs as a lead-free piezoelectric systems have attracted much attention because of their high Curie temperatures (*T*_c_), large spontaneous polarizations (*P*_s_), and good antifatigue properties [4,5,6,7,8]. The crystal structure of BLSFs consist of [Bi_2_O_2_]^2+^ layers interleaved with pseudo-perovskite [A_m−1_B_m_O_3m+1_]^2−^ units stacked along the *c*-axis, which can be described by a general chemical formula of (Bi_2_O_2_)^2+^(A_m−1_B_m_O_3m+1_)^2−^, where A is mono-, di-, trivalent ions, or a mixture of them and B is tetra-, penta-, hexavalent ions, or a mixture of both, and the integer *m* is the number of BO_6_ octahedral layer, taking any of the values from 1 to 5. The stability of the ABO_3_-type perovskite structure can be described by the tolerance factor (*t*), which can be expressed as [9]:

(1) $$t=\frac{r_{A}+r_{o}}{\sqrt{2}(r_{B}+r_{o})}$$

where *r_A_*, *r_B_* and *r_o_* are the ionic radius of A, B and the oxygen ion, respectively. The perovskite structure remains stable when *t* is between 0.77 and 1.10. However, the value of tolerance factor may be more limited in BLSF due to the structural instability between the pseudo-perovskite layer and the bismuth-oxygen layer, which is caused by the mismatching of their transverse dimension. Subbarao [10] proposed that when *m* values 2, 4 and 5, *t* is limited to 0.81~0.93, 0.85~0.89 and 0.86~0.87, respectively. This is to say, the range of the tolerance factor (*t*) tends to shrink with an increasing the number of octahedral layers (*m*).

**Figure 1:**
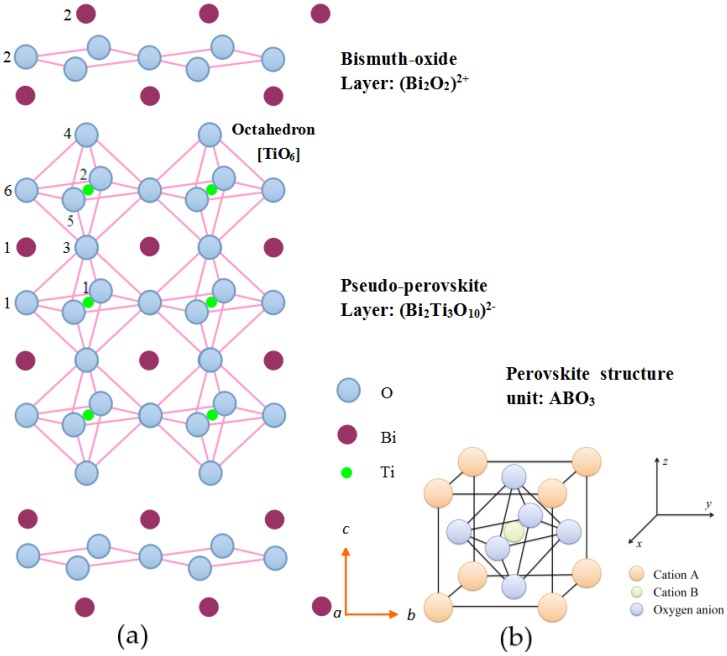
(**a**) A perspective drawing of Bi_4_Ti_3_O_12_ along [110] of the undistorted *Fmmm* parent structure (between *c*/4 and 3*c*/4 of unit cell); (**b**) A schematic sketch of perovskite structure unit.

Bismuth titanate, Bi_4_Ti_3_O_12_ (BIT) is the prototype of many bismuth layer structure oxides with the simplest chemical composition (*m* = 3). In Figure 1, its crystal structure can be described as a regular stacking of fluorite-like [Bi_2_O_2_]^2+^ slabs and perovskite-like [Bi_2_Ti_3_O_9_]^2−^ blocks, where three sheets of corner-sharing TiO_6_ octahedra make up this ABO_3_-type perovskite unit. This structure has two sites containing Bi^3+^, with different coordination environments. The [Bi_2_O_2_] layer site (Bi_2_) is an 8-coordinate position, whilst the perovskite A-site (Bi_1_) adopts 12-coordination. Also, there are two Ti^4+^ containing sites, namely, the perovskite B-sites, which are both distorted octahedral sites with the octahedra being rotated around the principal axis of unit cell. It is these octahedral distortions, together with the co-operative displacement of the A-site Bi_1_ atoms, that are considered to be the key to the ferroelectric properties of BIT [11]. Moreover, besides rotation, TiO_6_ octahedrons tend to incline away from the *c-*axis. As the result, the polarization measurements of BIT single crystals have shown a *P*_s_ of 50 μC/cm^2^ along the *a*-axis and of 3–5 μC/cm^2^ along the *c-*axis [12].

Armstrong and Newnham [13] studied the solubility limit of different ions doped in Bi_4_Ti_3_O_12_, and found that the estimated value of the lattice parameter (*a_B_*) of the unconstrained bismuth-oxygen layer was 3.89 Å, while that of the unconstrained pseudo-perovskite layer was 3.80 Å. However, the measured value of the lattice parameter (*a_T_*) of tetragonal Bi_4_Ti_3_O_12_ was 3.83 Å. These findings revealed a structural mismatch existing between the pseudo-perovskite layer and the bismuth-oxide layer of BIT. On the other hand, they also summarized that bismuth ions at the A-site (i.e., the apex of perovskite unit) could be substituted by the low-valence (+1⎕+3) metallic ions with an ionic radius between 1.1 Å and 1.3 Å, while titanium ions at the B-site (i.e., the center of oxygen-octahedron) could be replaced by the high-valence (+4⎕+6) metallic ions with a radius varying from 0.58 Å to 0.65 Å. However, in either event, the lower limit of doping-ions radii was determined by the stability of the pseudo-perovskite layer, while its upper limit depended on the mismatch degree between the pseudo-perovskite layer and the bismuth-oxide layer. Here, the tolerance factor of BIT (*m* = 3) was calculated to be 0.87, according to Equation (1). In the case of some commonly-used ions [13] doping into the A- or B-site of BIT, this factor was in the range of 0.86–0.92, which is just between *m* = 2 (0.81–0.93) and *m* = 4 (0.85–0.89).

In our previous work [14,15,16,17], we attempted a co-substitution of W^6+^ and Cr^3+^ for Ti^4+^ at the B-site of Bi_4_Ti_3_O_12_. This type of W/Cr co-doped Bi_4_Ti_3_O_12_ compound gained many good electrical and mechanical properties in the form of ceramics, becoming a very competitive candidate for high-temperature piezoelectric applications. Even though these doping-ions could keep the structural stability of pseudo-perovskite units, considering that their different bonding properties involved oxygen atoms, the structural matching relationship between the pseudo-perovskite layer and the bismuth-oxide layers might be changed by the lattice misfit that is induced [18]. Here, the lattice distortion of the whole structure could be adjusted by the induced lattice stress. Kikuchi [19] investigated the structural mismatch between the pseudo-perovskite layer and the bismuth oxide layer in the pseudo-tetragonal cell of Bi_3_TiNbO_9_ (*m* = 2). It was also suggested that such structural distortion may influence the phase stability, transformation characteristics, and polarization behavior of perovskite-type ferroelectrics [20,21].

However, to date, there are very few investigations with respect to these important problems in the doping system of Bi_4_Ti_3_O_12_. In this paper, both the lattice distortion and the interlayer mismatch were investigated for the Bi_4_Ti_3_O_12_ structure with W^6+^/Cr^3+^ co-doping, based on the stress analysis using an elastic model. The regulating rule of doping-ions for the structural deformation of bismuth layer structure oxides revealed by this work will help us to further understand the evolution of their properties.

## 2. Experimental Procedure

### 2.1. Sample Preparation

**Figure 2:**
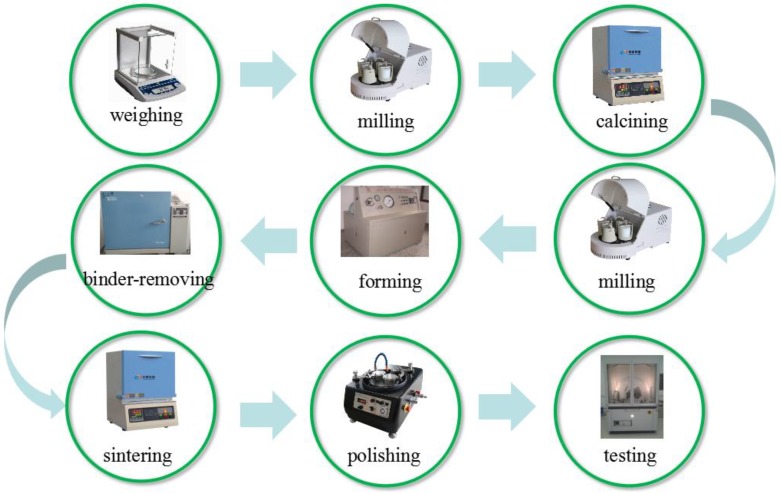
A conventional solid reaction process designed for synthesizing BTWC ceramics.

Aurivillius phase ceramics of Bi_4_Ti_3−x_W_x_O_12+x_ + y wt % Cr_2_O_3_ with 0 ≤ x ≤ 0.1 and 0 ≤ y ≤ 0.4, abbreviated as BTWC ceramics in Table 1, were synthesized via a conventional solid reaction process, as shown in Figure 2, using stoichiometric quantities of Bi_2_O_3_ (99.9%), TiO_2_ (98%), WO_3_ (99.9%) and Cr_2_O_3_ (99.9%). The detailed processing steps have been described in our previous works [14,15,16,17].

### 2.2. Phase Structure Analysis

The sintered BTWC ceramics were crushed into powder in an agate mortar and suspended in ethanol. The phase structures of powders were checked using an X-ray powder diffractometer (XRPD, Empyrean, PANalytical Co. Ltd., Almelo, The Netherlands) using CuKα radiation, in the 2θ range 10°–60°, with a step size of 0.02° (counting time was 15 s per step). The XRPD experimental diffraction patterns were analyzed via the Rietveld profile method using the MAUD program (Version: 2.7, written in Java by Luca Lutterotti, freely available his website). 

## 3. Results

### 3.1. Phase Structure of BTWC Ceramics

**Figure 3:**
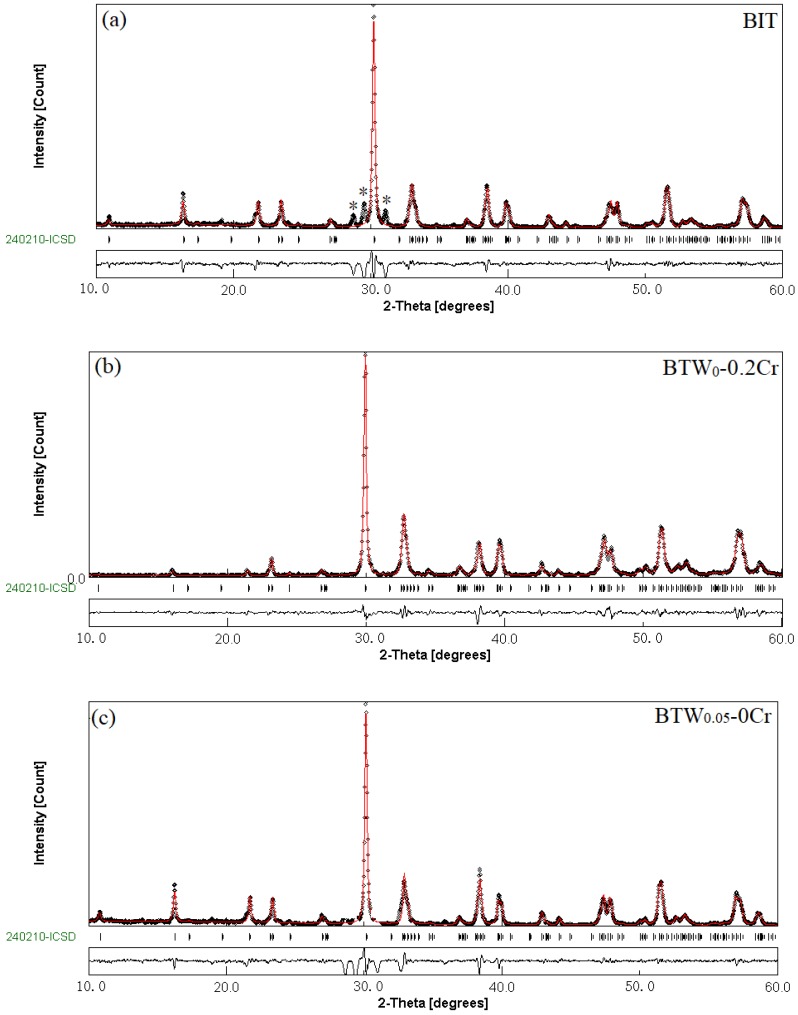

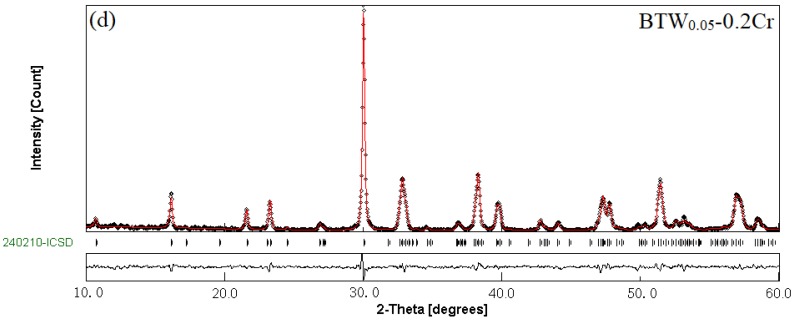
The observed (black circles), calculated (red curves), Bragg peaks position (vertical curves) and difference plots (obs-cal, black baselines) for the fit to the XRPD patterns of some BTWC ceramics after Rietveld refinement of the atomic structure at 295 K. (**a**) BIT; (**b**) BTW_0-_0.2Cr; (**c**) BTW_0.05-_0Cr; (**d**) BTW_0.05-_0.2Cr.

For a more quantitative assessment of the synthesis process on the phase structure of materials, XRPD pattern of some BTWC ceramics were registered at 295 K, as shown in Figure 3. In Figure 3a, all sets of reflections except for those specific ones noted by an asterisk could be indexed in the orthorhombic structure of the parent compound Bi_4_Ti_3_O_12_ (JCPDS card No. 72-1019), with a space group of *B*2*cb*(41). However, such impurity phases are most probably associated to the Bi_2_O_3_, TiO_2_ rutile and non-stoichiometric Ti_2_O_3_, which would be destroyed during longer milling time, without notable influence on the increased amount of the crystalline phase. As can be observed from Figure 3b–d, there seems to be very little difference in the patterns of BIT and W and/or Cr doped BIT. However, there is a slight shift of the reflections to lower 2θ values after the incorporation of W/Cr. According to the XRPD data, the Rietveld Refinement technique was used to calculate the parameters of the the pure Bi_4_Ti_3_O_12_ structure (Figure 3a) as follows, *a* = 5.4457 Å; *b* = 5.4087 Å; *c* = 32.8378 Å; *V* = 967 Å^3^ and *a*/*b* = 1.0068 (Refinement index: *R*_p_ = 5.67; *R*_wp_ = 7.42; *R*_B_ = 4.74; and λ^2^ = 1.37), which are basically in agreement with the values determined by Ivanov et al. [22]. Here, the factor with the form of *a*/*b* was used to indicate the degree of the lattice distortion of orthorhombic structure (ab. orthorhombic distortion), where a slight difference between the lattice constant of *a* and *b* was included.

### 3.2. Cell Parameters of BTWC Ceramics

The results from Rietveld Refinement for the crystal structures of BTWC ceramics are listed in Table 2 and Table 3, respectively. For BTW_x_-0.2Cr, the clear decrease of the orthorhombic distortion with increase in W content (x ≥ 0.025) correlates with the cationic polar displacements from ideal positions and the tilting adjustment of oxygen-octahedron. Thus BTW_0.05_-0Cr (only doped with W) shows a remarkable decrease of the orthorhombic distortion, as compared to that of BIT. The lattice model of octahedral distortion and the corresponding regulatory mechanism of doping ions will be discussed for BIT in Section 4.1.3.

## 4. Discussion

### 4.1. Lattice Distortion of Bi_4_Ti_3_O_12_ Structure

#### 4.1.1. Rotation of Oxygen-Octahedron

**Figure 4:**
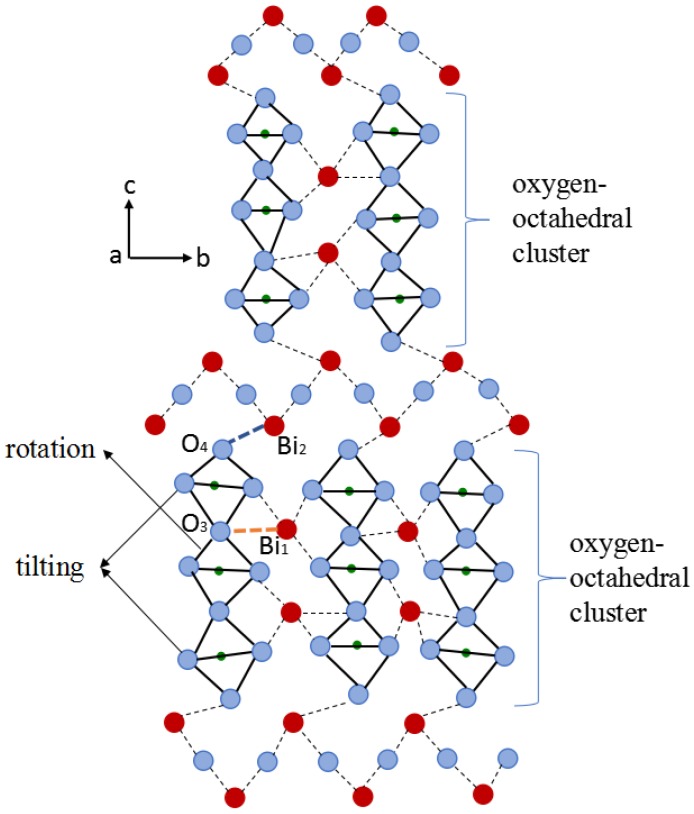
Cutaway drawing of Bi_4_Ti_3_O_12_ structures viewed along [100]. Octahedra (like rhombus) are outlined in solid lines, and dashed lines indicate the strong Bi–O bonds.

Figure 4 shows the cutaway drawing of Bi_4_Ti_3_O_12_ structures viewed along [100]. In this pattern, a lattice distortion is observed for the pseudo-perovskite layer of BIT which is constructed by the cluster of oxygen-octahedron. Here, a shear force induced by the shorter and stronger Bi–O bond between bismuth atoms at A-site (Bi1) and oxygen atoms in octahedron (O_3_), drives the central octahedron to rotate along the polarization axis (i.e., *a*-axis), thus stabilizing the pseudo-perovskite layer. Moreover, those three horizontal octahedra tend to rotate with the same angle (about 4°), leading to the same displacement between octahedra and bismuth atoms [23]. Even this octahedral distortion contributes to the principal component of the spontaneous polarization of BIT (i.e., *P*_s,a_).

Further, Martin Kunz [24] suggested a length matching relationship (*a*_A_/*a*_B_) between A–O bond and B–O bond as the criterion for the rotation of octahedron in the perovskite structure, as follows,

(2) $$aA/aB=\sqrt{2}r_{\left(A-O\right)}/2r_{\left(B-O\right)}$$

where both *r*_(A–O)_ and *r*_(B–O)_ are the bond length of A–O bond and B–O bond, respectively. According to the geometrical features of perovskite structure, the theoretical length of A–O bond should be $\sqrt{2}r_{\left(A–O\right)}$ while B–O bond 2*r*_(B–O)_. Therefore, if $r_{\left(A–O\right)}/r_{\left(B–O\right)}\neq \sqrt{2}$, a lattice stress will be induced due to the mismatch of bond length, causing the spontaneous distortion of octahedra to relieve such a mismatch.

Based on this theory, if *a*_A_/*a*_B_ < 1, the A–O bond will be stretched, while the B–O bond will be compressed, because A-site atoms look smaller. Conversely, if *a*_A_/*a*_B_ > 1, the A–O bond is compressed while the B–O bond will be stretched, because A-site atoms look bigger. Here, the relationship of *a*_A_/*a*_B_ < 1 for BIT according to the length of chemical bonds [25] can be obtained. As a result, the octahedron will rotate around the bismuth atom at the A-site along the *a*-axis, for the sake of relieving the mismatch of bond length by stretching Bi–O bond. However, it is worty noting that the rotation angle must meet the principle for the valence bond sum of cations in the structure unit.

#### 4.1.2. Tilting of Oxygen-Octahedron

When the structure of BIT undergoes the phase transition from the paraelectric phase to the ferroelectric phase, a stronger Bi–O bond between the bismuth atom in the bottom of the bismuth-oxide layer and the oxygen atom in the top of pseudo-perovskite layer will be produced to ensure the stability of the structure. Such a strong chemical bond tends to pull the two apical oxygen-octahedra, leading them to tilt along the *c*-axis. According to the description in [26], if choosing to define the octahedral tilts explicitly with respect to the unit cell edges, octahedral tilts can be calculated from the locations of the apical oxygen ions in the direction of interest. 

However, the tilt angles are absolute tilts calculated from the apical oxygen ion positions along the *c*-axis with respect to the *ab*-plane. The tilt angles are within ~1° for the central and outer BO_6_ oxygen-octahedra across the series, but the octahedra are also strongly distorted. 

In addition, according to the global parameterization method (GPM) proposed by Thomas [27], for the perovskite structure with any symmetry system, the lattice strain with the symbol of 90°-α_pc_ (α_pc_: pseudo-cubic angle) will cause the tilting of octahedron, and the corresponding tilting angle can be evaluated by the following formula,

(3) $$V_{A}/V_{B}=6\cos^{2}\theta_{m}\cos\theta_{z}-1$$

where, *θ_m_* and *θ_z_* are the tilting angle related to the pseudo-cubic axes. *V_A_* is the volume of AO_12_ polyhedron and *V_B_* is the volume of BO_6_ polyhedron. *V_A_* and *V_B_* are determined by the bond length of A–O bond and B–O bond (*d*_(A–O)_ and *d*_(B–O)_), respectively. It is believed that the tilting of octahedron stems forms the finite rotation of octahedron. A-site ions still need to deviate from the centre to form a displacement to coordinate the rotation, which can ensure that B-site ions are located in the centre of octahedron. If *V_A_*/*V_B_* < 5, the octahedron will tilt.

It was reported that *V*_Ca_/*V*_Ti_ = 4.61 in Ref. [28]. For the BIT with the orthorhombic structure, the value of *V*_Bi_/*V*_Ti_ may be smaller than 4.61, due to the stronger combining ability between Bi^3+^ and O^2−^ leading to the shorter Bi–O bond. This is because they have the similar ionic radius, but the electronegativity of Bi (2.02) is twice that of Ca (1.00). Therefore, the oxygen-octahedron of BIT is about to tilt, except for rotation. In addition, the first-principle calculations have shown a large cooperative coupling of Jahn-Teller (JT) distortion to oxygen-octahedron rotations in perovskite LaMnO_3_. The combination of the two distortions is responsible for stabilizing the strongly orthorhombic *A*-AFM insulating (*I*) *Pbnm* ground state, relative to a metallic ferromagnetic (FM-*M*) phase.

#### 4.1.3. Regulation of Doping-Ions for the Lattice Distortion

**Figure 5:**
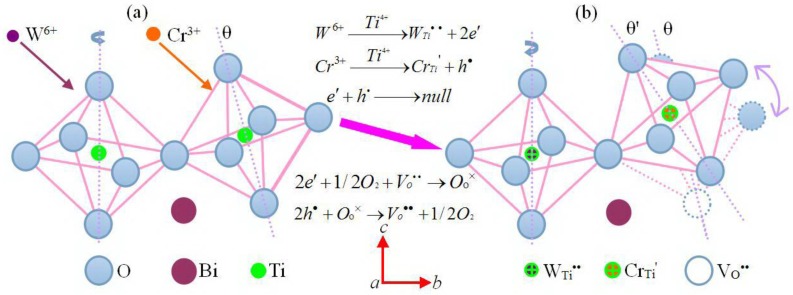
Status change of oxygen octahedron in Bi_4_Ti_3_O_12_ structure, (**a**) before W/Cr co-doping; (**b**) after W/Cr co-doping.

As analyzed above, the rotation and tilting of oxygen-octahedron within the pseudo-perovskite layer cause a slight difference in the length between *a*-axis and *b*-axis for Bi_4_Ti_3_O_12._ Based on the deformation mechanism of oxygen-octahedron, the status change of oxygen-octahedron in Bi_4_Ti_3_O_12_ structure after W/Cr co-doping can be described by the lattice model built in Figure 5. Here, the introduction of W^6+^ was performed in the calcining process of starting materials including WO_3_, while the incorporation of Cr^3+^ was addressed during sintering of the green bodies with the addition of Cr_2_O_3_. In view of their similar ionic radius (W^6+^: 0.600 Å, Cr^3+^: 0.615 Å and Ti^4+^: 0.605 Å) and same coordination number (6), both W^6+^ and Cr^3+^ are considered to substitute for Ti^4+^ in the core of oxygen-octahedron (i.e., B-site of perovskite structure). The difference in their bonding properties involved with oxygen atoms is likely responsible for the status change of oxygen-octahedron, which will be analyzed as follows.

Rietveld refinements of XRD have been used to calculate the value of orthorhombic distortion (*a*/*b*) for these BIT-based compounds. For BTW_x_-0.2Cr (Table 2), the orthorhombic distortion seems to linearly decrease with an increasing W content (*x*) from 0.025. Since the electronegativity of W (2.36) is larger than that of Ti (1.54), the length of W–O bond may be shorter than that of Ti–O bond within the octahedron. When W^6+^ occupies the B-site instead of partial Ti^4+^, the average value of *a*_A_/*a*_B_ will have an increase getting closer to 1, which tends to relieve the rotation of oxygen-octahedron according to Equation (2). Further evidence for this mechanism is derived from the composition of BTW_0.05_-0Cr, which was only doped by W^6+^, with its *a/b* value (1.0023) being much lower than that of BIT (1.0068). On the other hand, for BTW_0.05_-yCr (Table 3), Cr as a substitute has an approximatively equal electronegativity (1.66) as compared with Ti (1.54), which will not change the length of B–O bond significantly. Thus, the rotation status of oxygen-octahedron could remain constant, according to Equation 2. However, the substitution of Cr^3+^ for Ti^4+^ tends to create oxygen vacancy (V_O_^••^) in the oxygen-octahedron (as represented by the white circle in Figure 5), based on the principle of charge compensation [29], which has a significant influence on the volume of BO_6_ octahedron. As the result, this effect will change the tilting status of oxygen-octahedron, according to Equation 3. Finally, the value of *a/b* first increases, and then decreases with an increase of the amount of Cr_2_O_3_ (*y*), which can be considered as the synergistic reaction of these two regulatory mechanisms. It has been reported that the ferroelectric pseudo-perovskite of BLSF, with a higher degree of lattice distortion, usually possesses a larger spontaneous polarization in single ferroelectric domain [30]. As for W/Cr co-doped Bi_4_Ti_3_O_12_, the compositions with a higher orthorhombic distortion have also been shown to possess a larger spspontaneous polarization in polycrystalline ceramics, which was reported in one of our previous works [14,15].

### 4.2. Interlayer Mismatch of Bi_4_Ti_3_O_12_ Structure

#### 4.2.1. Elastic Model Considering Short Range Forces

Due to the rotation and tilting of oxygen octahedron, the pseudo-perovskite layer is distorted, which interferes the configuration of the bismuth-oxide layer. The structural mismatch between the pseudo-perovskite layer and the bismuth-oxide layer can be explained by the elastic strain energy [19]. Elastic model is a general method to deal with the lattice mismatch in epitaxial growth. Based on this model, the interlayer mismatch problem in the structure of Bi_4_Ti_3_O_12._ can be addressed.

It is supposed that the tetragonal structure of BIT has a lattice parameter of *a_T_*. Thus the relationship of lattice parameter between orthorhombic structure and tetragonal structure can be expressed as follows:

(4) $$a_{T}=(a_{orth}+b_{orth})/2\sqrt{2}$$

If half a unit cell is taken into consideration, the cell volume, *V*, can be expressed as follows:

(5) $$V=V_{B}+3V_{p}$$

where *V_B_* is the volume of bismuth-oxide layer unit and *V_P_* is that of pseudo-perovskite layer unit. Assuming that both the two units are elastomer, and then the internal stresses induced in the structure are expressed as:

(6a) $$F_{B}=-K_{B}({V^{\prime }}_{B}-V_{B})/{V^{\prime }}_{B}$$

(6b) $$F_{P}=-K_{P}({V^{\prime }}_{P}-V_{P})/{V^{\prime }}_{P}$$

where *K_B_* and *K_p_* are the bulk modulus, *V_B_* and *V_P_* are the volume of the constrained bismuth-oxide layer unit and pseudo-perovskite layer unit, respectively, *V_B_*’ and *V_p_*’ are the volume of the unconstrained units. (*V*’ − *V*)/*V* is defined as the volume strain (*ε*). When the two units are settled in the space lattice, *F_B_* and *F_P_* can be affected by not only the short range forces within the units but also the long range forces outside of the units, i.e., Madelung forces. In order to simplify the problem, only the short range forces are considered in this model.

Here, the unit volume of the tetragonal structure can be expressed as follows:

(7) $$V=a^{2}c$$

For the tetragonal structure, Equations (6a) and (6b) can be rewritten as follows:

(8a) $$F_{B}=-K_{B}(1-{a_{T}}^{2}c_{B}/{a^{\prime }}_{B}^{2}{c^{\prime }}_{B})$$

(8b) $$F_{P}=-K_{P}(1-{a_{T}}^{2}c_{P}/{a^{\prime }}_{P}^{2}{c^{\prime }}_{P})$$

Here, both (*a_B_*’, *c_B_*’) and (*a_P_*’, *c_P_*’) are the lattice parameters of the unconstrained bismuth oxide layer and pseudo-perovskite layer, respectively. For half an unit cell of BIT, a relationship between lattice parameters is expressed as:

(9) $$c_{B}+3c_{P}=c/2$$

The mismatch theory proposed by Armstrong and Newnham [13] pointed out the relationship that for *a_B_*’ < *a* < *a_P_*’ there is a lattice strain existing in the *ab-*plane, which is normal to the *c*-axis. Especially, under the biaxial compression or tensile load, the elastic responses of the bismuth oxide layer and pseudo-perovskite layer are similar in both tetragonal crystal and cubic crystal. Since the lattice strain tends to be distributed in the *ab-*plane because of the interval and alternation of two layers along the *c*-axis, the volume change in a unit cell can be considered without taking into account the length change of *c*-axis. If we consider the change of *a*-axis only, Equations (8a) and (8b) can be further simplified as follows:

(10a) $$F_{B}=-K_{B}(1-{a_{T}}^{2}/{a^{\prime }}_{B}^{2})$$

(10b) $$F_{P}=-K_{P}(1-{a_{T}}^{2}/{a^{\prime }}_{P}^{2})$$

**Figure 6:**
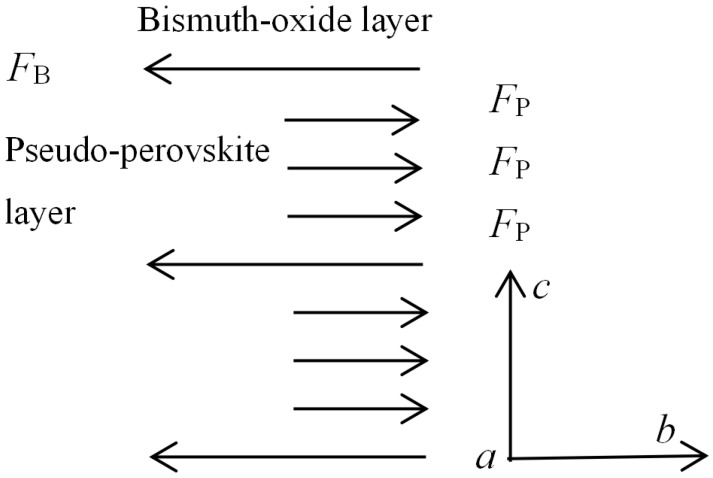
An analytic model for the plane stress distribution in the crystal lattice of Bi_4_Ti_3_O_12._

For a true crystal lattice of Bi_4_Ti_3_O_12_, the structure cannot be stable unless *F*_B_ and *F*_P_ not only act in the opposite manner, but also remain balanced with each other. Thus, an analytical model for the plane stress distribution in the crystal lattice of Bi_4_Ti_3_O_12_ could be constructed using the constitutive relationship of the alternating layer structure, as shown in Figure 6. 

According to the balance of forces, it yields:

(11) $$F_{B}=3F_{P}$$

Substituting Equations (10a) and (10b) into Equation (11), we can obtain:

(12) $$K=K_{P}/K_{B}=-(1-{a_{T}}^{2}/{a^{\prime }}_{B}^{2})/3(1-{a_{T}}^{2}/{a^{\prime }}_{P}^{2})$$

where *K* is defined as the interlayer mismatch degree, which is mainly determined by *K_P_* since *K_B_* is irrelevant to the composition of pseudo-perovskite layer. This is a confirmed structure and uniform composition for the bismuth-oxide layer of all BLSFs. *a_B_*’ is about 3.78 Å [13]. *a_P_*’ is assumed to be equal to the average lattice parameter of perovskite unit, which can be calculated as follows:

(13) $${a^{\prime }}_{P}=1.33r_{B}+0.60r_{A}+2.36Å$$

where *r_A_* and *r_B_* are the radius of the ions at A-site (Bi^3+^: 1.34 Å) and B-site of the perovskite unit. Finally, the total strain energy (*E*) generated by the expansion of the bismuth-oxide layer and the compression of pseudo-perovskite layer can be calculated by the following formula:

(14) $$E=0.5K_{B}\Delta V$$

(15) $$\Delta V={V^{\prime }}_{B}{\left(1-{a_{T}}^{2}/{a^{\prime }}_{B}^{2}\right)}^{2}+3K{V^{\prime }}_{P}{\left(1-{a_{T}}^{2}/{a^{\prime }}_{P}^{2}\right)}^{2}$$

where *V_B_*’ = *a_B_*’^2^ *c_B_* ≈ 65.4 Å^3^, *V_P_*’ = *a_P_*’^3^. According to the principle of solid physics, the bulk modulus of ionic crystal is determined by the following formula [30]:

(16) $$K_{bulk}=\alpha e^{2}/{r_{0}}^{4}$$

Here, α is Madelung constant, which is 24.76 for A^II^B^IV^O_3_ [19]. *r*_0_ is the shortest distance between cations and anions, which is 1.73 Å for Bi_4_Ti_3_O_12_ (B = Ti) [23], and *e =* 4.8 × 10^−10^ esu. Hereby, *K_P_* can be approximatively calculated as 44.26 GPa, and *K_B_* can be also determined as 98.36 GPa according to the value of *K* (0.45) for Bi_4_Ti_3_O_12_ reported in Ref. [19].

#### 4.2.2. Influence of Doping-Ions on the Interlayer Mismatch

In this experiment, the chemical formula of BTWC ceramics is: Bi_4_Ti_3−x_W_x_O_12+x_ + y wt % Cr_2_O_3_ (0 ≤ x ≤ 0.1; 0 ≤ y ≤ 0.4). The part of titanium at the B-site of perovskite structure was co-substituted by the tungsten and chromium introduced by dopants. If the mass fraction of Cr_2_O_3_ is changed into the molar fraction relative to the chemical composition, the chemical formula of BTWC ceramics can be written as Bi_4_Ti_3−x−0.154y_W_x_Cr_0.154y_O_12+x−0.077y_. As the B-site is occupied by Ti^4+^/W^6+^/Cr^3+^ together, in order to simply the calculation, *r*_B_ is given by the average value of radius of all local ions (W^6+^ ~ 0.600 Å, Cr^3+^ ~ 0.615 Å and Ti^4+^ ~ 0.605 Å), in relation to their molar ratio:

(17) $$r_{B}=0.605\times \frac{3-x-0.154y}{3}+0.6\times \frac{x}{3}+0.615\times \frac{0.154y}{3}$$

Finally, according to Equations (13)–(15) obtained from the elastic model and lattice parameters derived from XRD, the interlayer mismatch degree (*K*) and the total strain energy (*E*) can be calculated for BTWC. 

**Figure 7:**
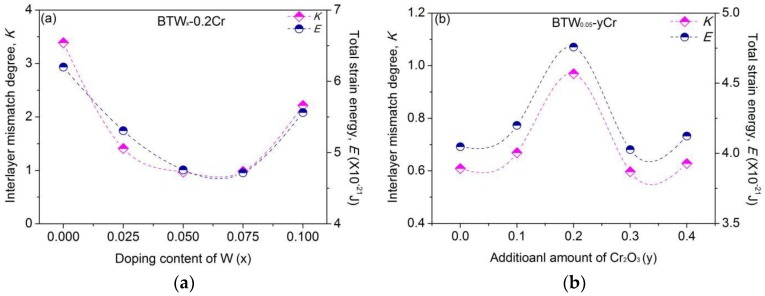
Variation of the interlayer mismatch degree and total strain energy of BTWC with the composition (**a**) varying with the doping content of W (*x*); (**b**) varying with the additional amount of Cr_2_O_3_ (*y*).

Figure 7 shows the variation of interlayer mismatch degree and total strain energy of BTWC with the composition. As can be seen, the interlayer mismatch degree shows a similar varying trend to the total strain energy. That is to say, a more mismatched layer structure stores a larger strain energy for a crystal. In addition, the calculated value of *K* is 0.48 for BIT in this paper, while BTW_x_-0.2Cr and BTW_0.05_-yCr take the values of *K* in the range of 0.97–3.45 and 0.60–0.97, respectively. It seems to be that either W^6+^ or Cr^3+^ can aggravate the interlayer mismatch of Bi_4_Ti_3_O_12_ structure. This may be partly due to the mechanism whereby doping of W^6+^/Cr^3+^ could relieve the distortion of pseudo-perovskite layer by depressing the rotation and tilting of oxygen octahedron (see the values of *a/b* in Table 2 and Table 3), which helps the pseudo-perovskite layer to be more stiff.

**Figure 8:**
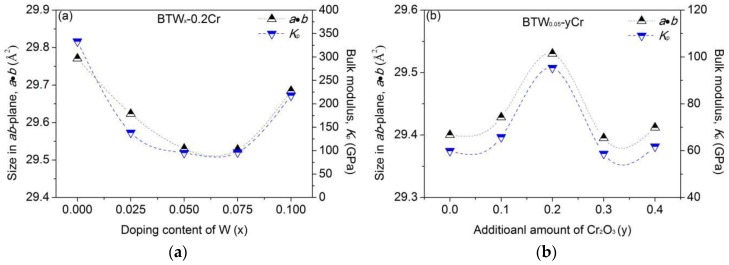
Variation of the size in *ab-*plane and the bulk modulus of perovskite unit of BTWC with the composition (**a**) varying with the doping content of W (*x*); (**b**) varying with the additional Cr_2_O_3_ (*y*).

However, in fact, the interlayer mismatch degree should be more dependent on the size in *ab-*plane for the Bi_4_Ti_3_O_12_ structure, which is illuminated by Figure 8. Also, the variation of the size in the *ab-*plane with the dopant content is consistent with that of the bulk modulus of the perovskite unit, which indicates that a larger perovskite unit tends to possess a higher bulk modulus. Above all, for all the compositions of BTWC, the size in *ab-*plane keeps the same varying tendency with the interlayer mismatch degree. The results can strongly prove that the interlayer mismatch in Bi_4_Ti_3_O_12_ structure has to be constrained in the *ab-*plane of the unit cell. In general, with larger size of *ab-*plane, a higher bulk modulus of perovskite unit is achieved; then, a larger interlayer mismatch of Bi_4_Ti_3_O_12_ structure, as well as a higher strain energy stored in the crystal occur.

## 5. Conclusions

For some W/Cr co-doped Bi_4_Ti_3_O_12_ (ab. BTWC) Aurivillius compounds, XRD analysis shows that all compositions of BTWC belong to the orthorhombic structure, and Rietveld refinements for the crystal structure are used to calculate their lattice constants. Since the oxygen-octahedron rotates in the *ab-*plane, as well as inclines away from the *c-*axis, W^6+^ could occupy the B-site instead of partial Ti^4+^, which tends to relieve the rotation of oxygen-octahedron. The substitution of Cr^3+^ for Ti^4+^ tends to create an oxygen vacancy (V_O_^••^) in the oxygen-octahedron, which has an influence on the tilting of oxygen-octahedron. Due to the strong combining power between the pseudo-perovskite layer and the bismuth oxide layer along the *c*-axis, a lattice misfit tends to be constrained to the *ab-*plane, which causes a structural mismatch between the two layers. Both the degree of interlayer mismatch and the total strain energy vary with the compositions of BTWC in a similar trend to the lattice parameters in the *ab-*plane. The doping of W^6+^/Cr^3+^ could relieve the distortion of pseudo-perovskite layer of BIT, which helps it to be more stiff.

## Figures and Tables

**Table 1:** Composition design and sample number of Bi_4_Ti_3−x_W_x_O_12+x_ + y wt % Cr_2_O_3_ (BTWC) ceramics.

	x	0	0.025	0.05	0.075	0.1
y						
0		BIT	-	BTW_0.05_-0Cr	-	-
0.1		-	-	BTW_0.05_-0.1Cr	-	-
0.2		BTW_0_-0.2Cr	BTW_0.025_-0.2Cr	BTW_0.05_-0.2Cr	BTW_0.075_-0.2Cr	BTW_0.1_-0.2Cr
0.3		-	-	BTW_0.05_-0.3Cr	-	-
0.4		-	-	BTW_0.05_-0.4Cr	-	-

**Table 2:** Rietveld refinements for the crystal structure of BTW_x_-0.2Cr at 295 K.

Parameters	Tungsten Content in BTW_x_-0.2Cr (Space Group of *B*2*cb*)				
	*x* = 0	*x* = 0.025	*x* = 0.05	*x* = 0.075	*x* = 0.1
**Refinement index**					
*R*_p_	5.68	5.59	5.54	5.70	5.62
*R*_wp_	7.38	7.29	7.25	6.61	7.33
R_B_	4.99	4.83	5.54	5.41	4.85
λ^2^	1.35	1.41	1.44	1.28	1.31
**Lattice parameters (Å)**					
*a*	5.4699	5.4578	5.4485	5.4464	5.4567
*b*	5.4427	5.4277	5.4199	5.4215	5.4400
*c*	32.9354	32.9155	32.8742	32.8320	32.9274
*V* (Å^3^)	982	975	971	969	979
Orthorhombic distortion (*a*/*b*)	1.0050	1.0063	1.0053	1.0046	1.0031

**Table 3:** Rietveld refinements for the crystal structure of BTW_0.05_-yCr at 295 K.

Parameters	Chromium Content in BTW_0.05_-yCr (Space Group of *B*2*cb*)				
	*y* = 0	*y* = 0.1	*y* = 0.2	*y* = 0.3	*y* = 0.4
**Refinement index**					
*R*_p_	5.68	5.59	5.54	5.70	5.62
*R*_wp_	7.38	7.29	7.25	6.61	7.33
R_B_	4.99	4.83	5.54	5.41	4.85
λ^2^	1.35	1.41	1.44	1.28	1.31
**Lattice parameters (Å)**					
*a*	5.4285	5.4341	5.4485	5.4344	5.4334
*b*	5.4159	5.4155	5.4199	5.4091	5.4132
*c*	32.8595	32.8796	32.8742	32.8516	32.8551
*V* (Å^3^)	966	968	971	967	966
Orthorhombic distortion (*a*/*b*)	1.0023	1.0034	1.0053	1.0047	1.0037
